# CD140b and CD73 are markers for human induced pluripotent stem cell‐derived erythropoietin‐producing cells

**DOI:** 10.1002/2211-5463.12800

**Published:** 2020-02-13

**Authors:** Shogo Nishimoto, Tomohiro Mizuno, Kazuo Takahashi, Fumihiko Nagano, Yukio Yuzawa, Akira Nishiyama, Kenji Osafune, Hirofumi Hitomi, Tadashi Nagamatsu

**Affiliations:** ^1^ Department of Analytical Pharmacology Meijo University Nagoya Japan; ^2^ Department of Nephrology School of Medicine Fujita Health University Toyoake Japan; ^3^ Department of Pharmacology Faculty of Medicine Kagawa University Japan; ^4^ Department of Cell Growth and Differentiation Center for iPS Cell Research and Application (CiRA) Kyoto University Japan; ^5^ Department of iPS Stem Cell Regenerative Medicine Kansai Medical University Osaka Japan

**Keywords:** CD140b, CD73, chronic kidney disease, erythropoietin, human induced pluripotent stem cells

## Abstract

Renal anemia in chronic kidney disease is treated with recombinant human erythropoietin (rhEPO). However, some patients with anemia do not respond well to rhEPO, emphasizing the need for a more biocompatible EPO. Differentiation protocols for hepatic lineages have been modified to enable production from human induced pluripotent stem cell (hiPSC)‐derived EPO‐producing cells (EPO cells). However, markers for hiPSC‐EPO cells are lacking, making it difficult to purify hiPSC‐EPO cells and therefore to optimize EPO production and cell counts for transplantation. To address these issues, we investigated whether CD140b and CD73 could be used as markers for hiPSC‐EPO cells. We measured the expression of EPO, CD140b, and CD73 in hiPSC‐EPO cells and the EPO concentration in the cell supernatant by immunohistochemistry and enzyme‐linked immunosorbent assays on culture day 13, revealing that expression levels of CD140b and CD73 are correlated with the level of EPO. In addition, rates of CD140b^+^ CD73^+^ cells were observed to be correlated with the concentration of EPO. Thus, our results suggest that CD140b and CD73 may be markers for hiPSC‐EPO cells.

## Graphical abstract

We established differentiation protocols for human induced pluripotent stem cell (hiPSC)‐derived erythropoietin‐producing cells (EPO cells). However, markers for hiPSC‐EPO cells are lacking, making it difficult to purify hiPSC‐EPO cells and therefore to optimize EPO production and cell counts for transplantation. Here, we elucidated that CD140b and CD73 may be markers for hiPSC‐EPO cells.

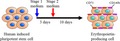

**Abbreviations**
CKD: chronic kidney disease
hiPSC: human induced pluripotent stem cell
KSR: KnockOut Serum Replacement
NCCLS: National Committee for Clinical Laboratory Standards
NEAA: nonessential amino acid
PBST: PBS/0.1% Triton X‐100
rhEPO: recombinant human erythropoietin

Erythropoietin (EPO) has an essential role in erythropoiesis 1. The kidney is the main organ for EPO production in adults; however, EPO production is severely reduced in patients with chronic kidney disease (CKD) 2. To improve renal anemia, patients with CKD are treated with recombinant human EPO (rhEPO). Although rhEPO improves renal anemia and mortality in patients with CKD, patients have to receive rhEPO infusions one to three times per week to maintain the erythropoiesis level 3, 4. In addition, it is necessary to consider the total cost of rhEPO.

A previous report has suggested that patients with anemia secondary to chronic diseases may not respond well to rhEPO 5. In very rare cases, patients with germline mutations in EPO 6 or with anti‐rhEPO autoantibodies after treatment with rhEPO have been reported 7, 8. To solve these problems, it is necessary to develop a more biocompatible EPO.

We have modified a previously reported differentiation protocol for hepatic lineages to establish a protocol for generating human induced pluripotent stem cell (hiPSC)‐derived EPO‐producing cells (EPO cells) 9. hiPSC‐EPO cells were more beneficial for the treatment of renal anemia in mice with CKD than rhEPO 9. However, for transplantation, over 1.0 × 10^7^ iPSC‐EPO cells per mouse were needed. In addition, markers for hiPSC‐EPO cells have not been identified; therefore, there are no methods for the purification of hiPSC‐EPO cells. To enable the application of hiPSC‐EPO cells in regenerative medicine, the identification of markers for hiPSC‐EPO cells and the purification of hiPSC‐EPO cells are needed to optimize EPO production and the number of hiPSC‐EPO cells for transplantation. Previous studies have suggested that CD140b and CD73 are markers for renal EPO cells 10, 11, 12. Since these proteins are expressed on the surfaces of cells, if they are also expressed in hiPSC‐EPO cells, a cell sorting approach can be used for isolation. In addition, hiPSC‐EPO cells are differentiated from hiPS cells cultured on feeder cells. To avoid xenotransplantation, a differentiation protocol from hiPSCs cultured by the nonfeeder culture system should be established. Accordingly, we investigated whether CD140b and CD73 are markers for EPO cells differentiated from hiPSCs obtained by a feeder‐free culture system.

## Methods

### hiPSC culture

The hiPSCs (253G1) were provided by the RIKEN BRC through the Project for Realization of Regenerative Medicine and the National Bio‐Resource Project of the MEXT, Japan. The hiPSCs were cultured under feeder‐free conditions according to a previous report 13. Briefly, hiPSCs were seeded on plates coated with iMatrix‐511 (Nippi, Tokyo, Japan). hiPSCs were incubated in StemFit medium supplemented with Y‐27632 (10 µm; Wako, Osaka, Japan) for the first 24 h, and the medium was replaced with StemFit medium without Y‐27632. The StemFit medium was replaced with fresh medium every other day until culture day 7. After day 7, hiPSCs were used for differentiation protocols to obtain EPO cells.

### Differentiation protocols for hiPSC‐EPO cells

**Figure 1:**
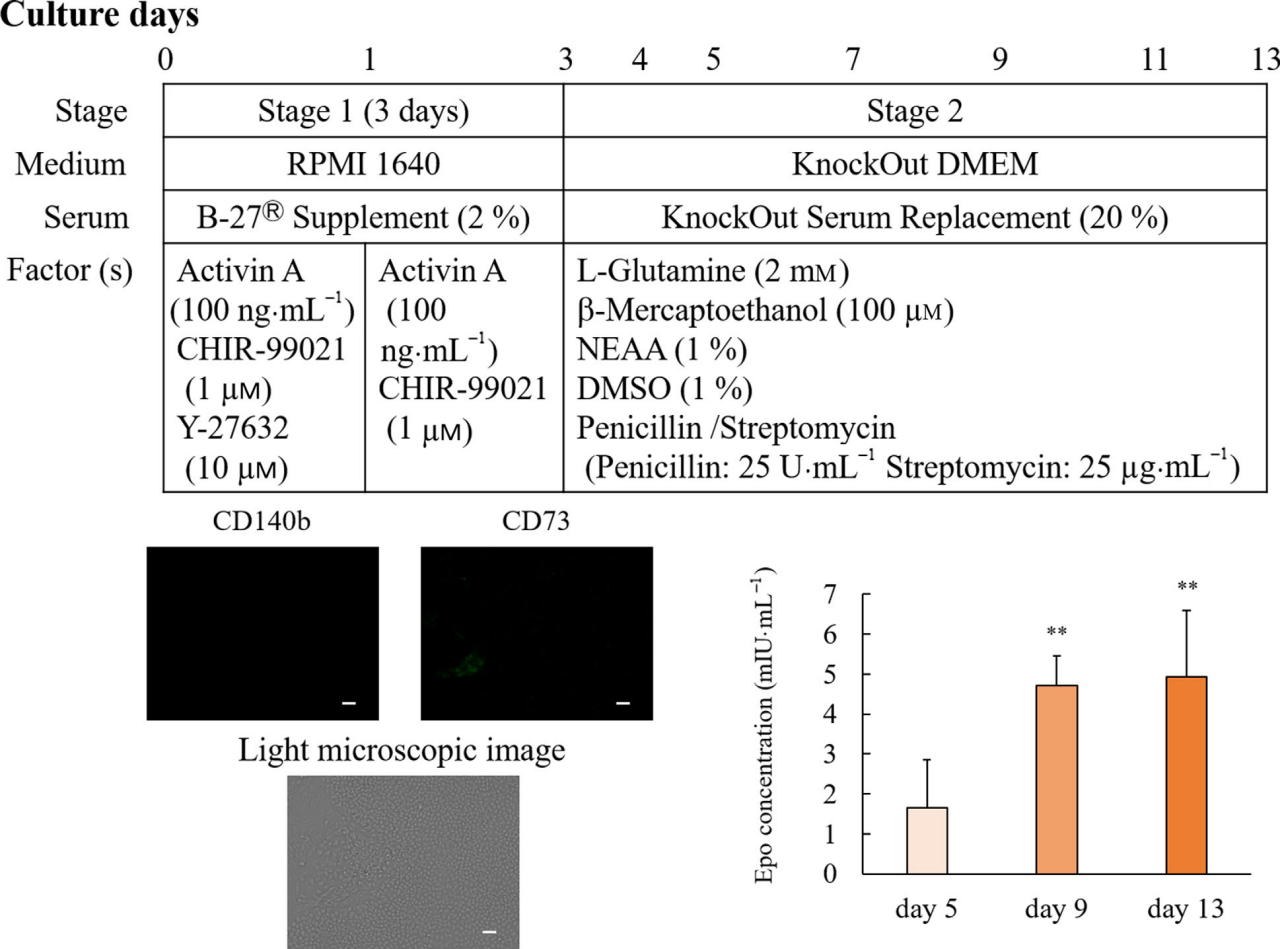
Erythropoietin concentration in the supernatant and overview of the modified differentiation protocol. The differentiation protocol was performed by modifying a previous method 9. hiPSCs grown on plates coated with iMatrix‐511 were cultured in StemFit medium for 7 days. The hiPSCs were dissociated to single cells by gentle pipetting after treatment with Accutase and seeded on Matrigel‐coated plates with stage 1 medium containing RPMI 1640 supplemented with B27 supplement, recombinant human/mouse/rat activin A, and 1 μm CHIR99021. After 24 h, this medium was replaced with fresh medium without Y‐27632 until culture day 3. The medium was changed to stage 2 medium containing KnockOut DMEM supplemented with penicillin/streptomycin, 20% KSR, 1% DMSO, 2 mm l‐glutamine, 1% NEAA, and 100 μm β‐mercaptoethanol. The hiPSCs were stained with anti‐CD140b (red color) and anti‐CD73 (green color) antibodies. The white bar indicates 100 µm. EPO concentrations are shown as mean values ± SD. **P < 0.01 vs. day 5 (Bonferroni's test).

The differentiation of EPO cells from hiPSCs was performed according to a previously reported protocol, with modifications (Fig. 1). hiPSCs harvested in StemFit medium were dissociated to single cells by gentle pipetting after treatment with Accutase (Innovative Cell Technologies, San Diego, CA, USA) for 2 min at 37 °C and seeded on Matrigel (Corning, Inc., Corning, NY, USA)‐coated plates at a density of 5.0 × 10^4^ cells·cm^−2^ with stage 1 medium containing RPMI 1640 (Nacalai Tesque, Kyoto, Japan) supplemented with B27 supplement (Thermo Fisher Scientific, Waltham, MA, USA), recombinant human/mouse/rat activin A (100 ng·mL^−1^) (PeproTech Inc., Princeton, NJ, USA), and 1 μm CHIR99021 (Wako). Y‐27632 (10 µm; Wako) was added to the stage 1 medium for the first 24 h. After 24 h, this medium was changed to the fresh medium without Y‐27632 until culture day 3. On culture day 3, the medium was changed to stage 2 medium containing KnockOut DMEM (Thermo Fisher Scientific) supplemented with penicillin/streptomycin (25 U·mL^−1^), 20% KnockOut Serum Replacement (KSR), 1% DMSO, 2 mm l‐glutamine, 1% nonessential amino acid (NEAA), and 100 μm β‐mercaptoethanol. Stage 2 medium was replaced every day until culture day 5. After culture day 7, stage 2 medium was replaced with fresh medium every other day until culture day 13.

### Immunocytochemistry

EPO, CD140b, and CD73 on hiPSC‐EPO cells, and CD140b and CD73 on hiPSCs were evaluated by immunocytochemistry. Immunostaining of EPO was performed according to previously described methods 9. In brief, hiPSC‐EPO cells were fixed with 4% paraformaldehyde/PBS for 20 min at 4 °C. After washing with PBS, the cells were permeabilized by PBST (PBS/0.1% Triton X‐100) for 20 min at room temperature (18–25 °C). To block Fc receptors, the cells were incubated with 3% TruStain FcX™ (Biolegend, San Diego, CA, USA) for 10 min at room temperature after permeabilization. The cells were then incubated with mouse anti‐human EPO antibody (Merck Millipore, Burlington, MA, USA) overnight at 4 °C followed by a conjugate of Alexa 488‐labeled polyclonal goat anti‐mouse IgG antibody (Biolegend). After washing, a PE‐labeled rat monoclonal anti‐CD73 antibody (Biolegend) or an APC‐labeled rat monoclonal anti‐CD140b antibody (Biolegend) was applied for 30 min at room temperature. After washing with PBS, images were captured at ×100 magnification using a BZ‐X700 Fluorescence Microscope (Keyence, Osaka, Japan). The hiPSC‐EPO cells treated with TruStain FcX™ were used as a background control, and then, the positive areas for EPO, CD140b, and CD73 were measured using Image Express (Molecular Devices, San Jose, CA, USA) in 20 sequential fields, and values were obtained.

### Fluorescent‐activated cell sorting

To quantify CD140b^+^ CD73^+^ cells, hiPSC‐EPO cells were dissociated to single cells by treatment with Accutase for 10 min at 37 °C. After washing with PBS, the cells were incubated with 3% TruStain FcX™ for 10 min at room temperature. Then, the cells were incubated with a PE‐labeled mouse monoclonal anti‐CD73 antibody and an APC‐labeled mouse monoclonal anti‐CD140b antibody for 30 min at room temperature. After washing with PBS containing 1% FBS, fluorescent‐activated cell sorting was performed using a BD LSR Fortessa X‐20 System (Becton Dickinson, Franklin Lakes, NJ, USA) to detect expressions of CD140b and CD73 on the cell surface.

### Enzyme‐linked immunosorbent assay

The concentrations of EPO in the medium were measured by ELISA according to the manufacturer's protocol (ALPCO Diagnostics, Salem, NH, USA). In brief, the culture medium of hiPSC‐EPO cells was added to a 96‐well plate with enzyme‐labeled anti‐human EPO antibodies and incubated for 2 h at room temperature. The substrate was then incubated on the plate for 30 min, and the reaction was stopped with 0.5 mm sulfuric acid. The plates were read at 450 nm using the iMark™ Microplate Reader (Bio‐Rad, Hercules, CA, USA), and the concentration of EPO was calculated on the basis of the standard curve for lyophilized synthetic EPO.

### Statistical analyses

Comparisons among multiple groups were conducted by one‐way ANOVA followed by Bonferroni's tests. A correlation analysis was performed by Pearson's (parametric parameters) or Spearman's (nonparametric parameters) methods. A two‐sided *P*‐value of < 0.05 was considered significant. spss v22.0 (SPSS, Chicago, IL, USA) was used for statistical analyses.

## Results

### hiPSC‐EPO cells secreted erythropoietin

We previously reported that EPO secretion from hiPSC‐EPO cells peaks on culture days 11–13 9. In the present study, hiPSCs were cultured by a feeder‐free culture system (Fig. 1) for differentiation to hiPSC‐EPO cells. The peak of EPO secretion by hiPSC‐EPO cells was detected on culture day 13. According to the National Committee for Clinical Laboratory Standards (NCCLS), the reference range for the EPO concentration in human serum is 3.22–31.9 mIU·mL^−1^. The levels exceeded 3.22 mIU·mL^−1^ on culture days 9 and 13, which were within this reference range.

### CD140b and CD73 were correlated with the concentration of erythropoietin

**Figure 2:**
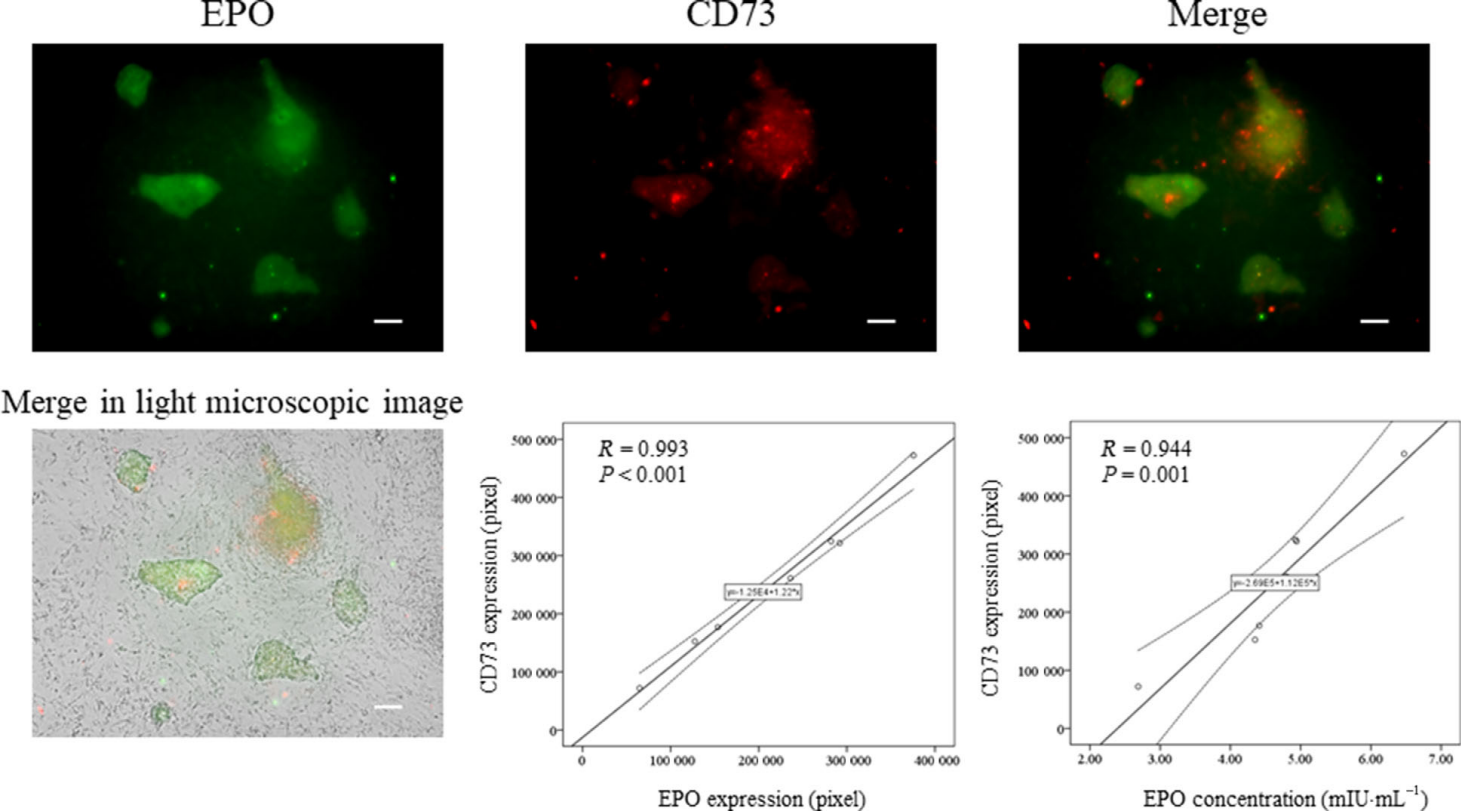
Correlation between EPO and CD73 expression. EPO and CD73 expressions were evaluated by immunocytochemistry. The hiPSC‐EPO cells were stained with anti‐EPO (green) and anti‐CD73 antibodies (red). The white bar indicates 100 µm. The *P*‐values were determined by Pearson's tests.

**Figure 3:**
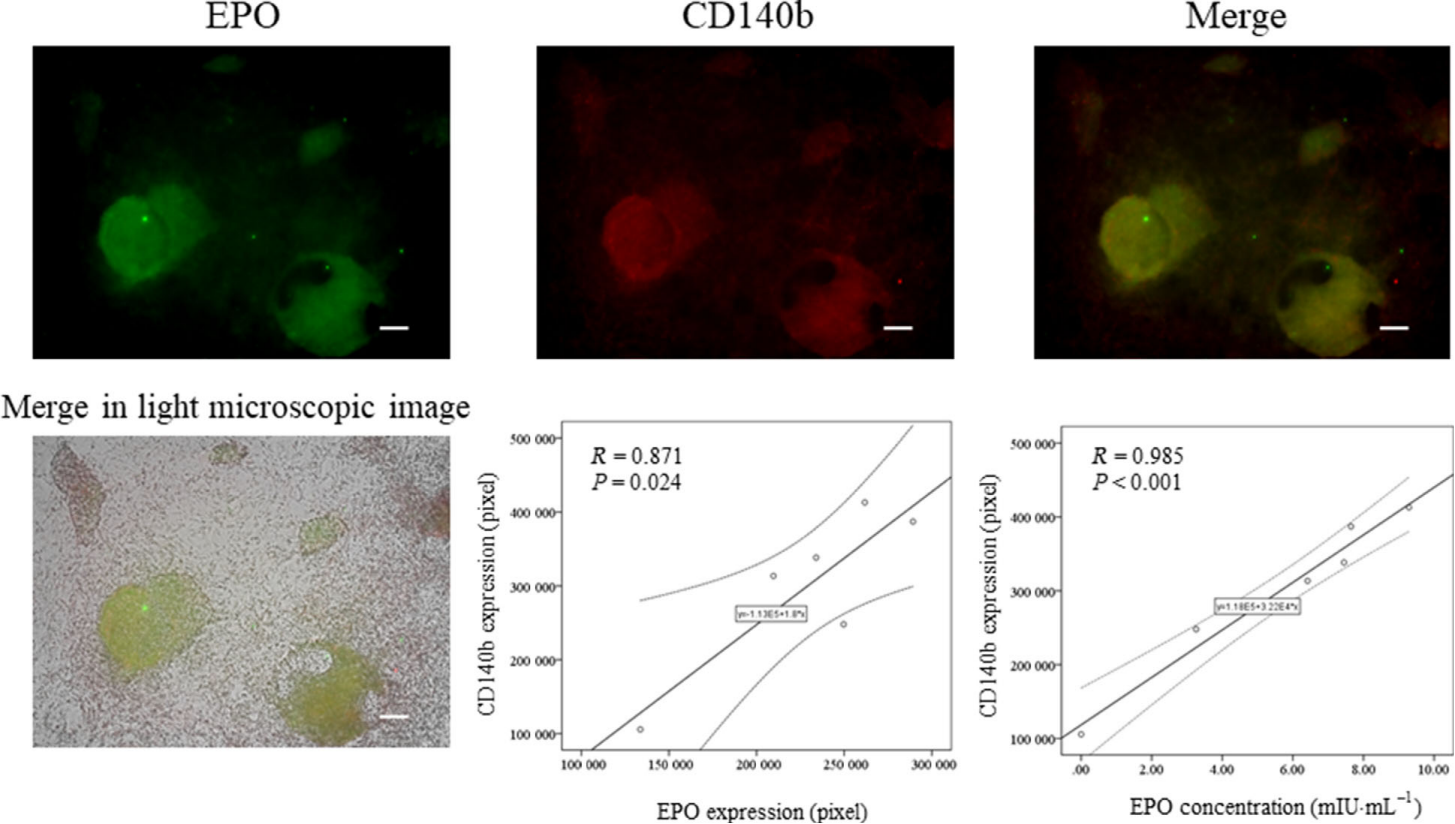
Correlation between CD140b and erythropoietin expression. EPO and CD140b expressions were evaluated by immunocytochemistry. hiPSC‐EPO cells were stained with anti‐EPO (green) and anti‐CD140b antibodies (red). The white bar indicates 100 µm. The *P*‐values were determined by Pearson's tests.

To investigate whether CD140b and CD73 could be markers for hiPSC‐EPO cells, we evaluated correlations between CD140b or CD73 and EPO on hiPSC‐EPO cells. The expressions of CD140b and CD73 on hiPSCs were negative (Fig. 1). The expression of CD73 was correlated with the expression of EPO (*R* = 0.993, *P* < 0.001) and the concentration of EPO in the cell supernatant (*R* = 0.944, *P* = 0.001) (Fig. 2). The expression of CD140b was correlated with the expression of EPO (*R* = 0.871, *P* = 0.024) and the concentration of EPO in the supernatant (*R* = 0.985, *P* < 0.001) (Fig. 3). These results suggested that CD140b and CD73 are markers for hiPSC‐EPO cells.

### Rates of CD140b^+^ CD73^+^ cells were correlated with the concentration of erythropoietin

**Figure 4:**
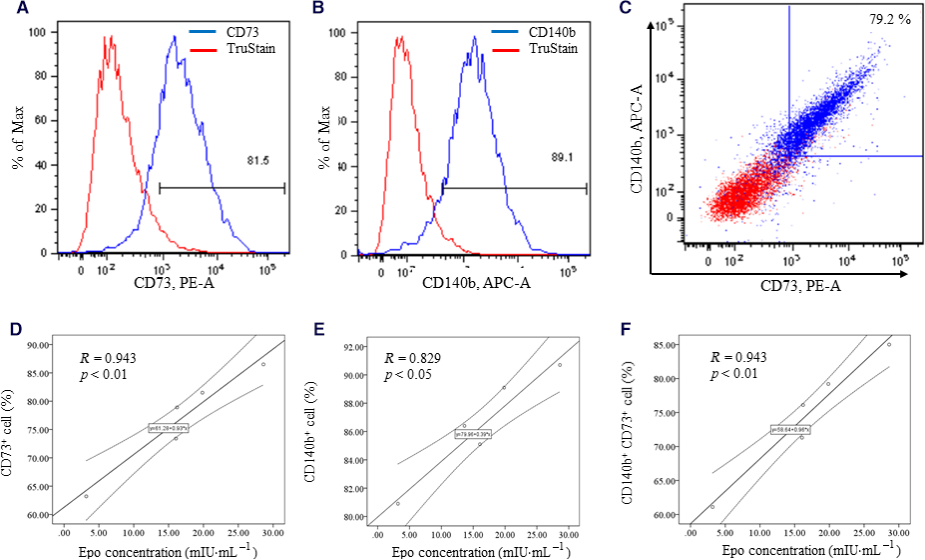
Correlation between CD140b and CD73 and erythropoietin secretion. The rates of positive cells were determined by the above gating strategy (A–C). The correlation between the rate of CD73^+^ cells and EPO concentration is shown in (D). The correlation between the rate of CD140b^+^ cells and EPO concentration is shown in (E). The correlation between the rate of CD140b^+^ CD73^+^ cells and EPO concentration is shown in (F). The *P*‐values were determined by Spearman's tests.

For the purification for hiPSC‐EPO cells, we evaluated the correlations between the rates of CD140b^+^ CD73^+^ cells and the concentration of EPO in the supernatant. The rates of CD140b^+^, CD73^+^, and CD140b^+^ CD73^+^ cells were correlated with EPO in the supernatant (*R* = 0.943, *P* < 0.01; *R* = 0.829, *P* < 0.05; *R* = 0.943, *P* < 0.01, respectively) (Fig. 4). These results suggested that CD140b and CD73 are targets for cell sorting.

## Discussion

We previously established a differentiation protocol for hiPSC‐EPO cells 9 using hiPSCs cultured by a feeder culture system. Although feeder cells are widely used to maintain and culture hiPSCs, the preparation of feeder cells is time‐consuming. In addition, to satisfy the Standard for Biological Ingredients, FBS‐containing medium should be removed entirely. We established a differentiation protocol for hiPSC‐EPO cells produced from hiPSCs cultured in feeder‐free conditions. We investigated whether hiPSC‐EPO cells generated by the new differentiation protocol can produce EPO and found that the EPO concentration was within the reference range recommended by the NCCLS. Although further studies are needed to verify these findings, they suggest that hiPSC‐EPO cells were successfully produced from hiPSCs in feeder‐free conditions.

In our previous report, hiPSC‐EPO cells ameliorated renal anemia in CKD mice; however, optimization of the number of hiPSC‐EPO cells needed purification. Recent reports have suggested that CD140b and CD73 are markers for EPO cells 10, 11, 12. Our previous differentiation protocol was established for hiPSCs/embryonic stem cells through hepatic lineage differentiation, and it was not clear whether hiPSC‐EPO cells have similar characteristics. In addition, markers for hiPSC‐EPO cells were lacking. Accordingly, we investigated whether hiPSC‐EPO cells express CD140b and CD73. In Fig. 4, the rates of CD140b^+^ cells are higher than those of CD140b^+^ CD73^+^ cells. This result indicated that CD73^‐^ cells would be included in CD140b^+^ cell. Moreover, the correlation coefficient between CD73^+^ cells and EPO secretion was higher than that of CD140b^+^ cells. To exclude CD140b^+^ CD73^‐^ cells, we evaluated the correlation between the rate of CD140b^+^ CD73^+^ cells and EPO secretion in the medium. The rates of CD140b^+^ CD73^+^ cells correlated with EPO secretion. These results suggested that CD140b^+^ CD73^+^ cells produce EPO. In addition, these proteins are expressed on cell surfaces. To remove CD140b^‐^ CD73^‐^ cells, which would be positive for hepatic lineage and endoderm markers 9, CD140b and CD73 might be useful for the purification by cell sorting. For the purification and optimization of hiPSC‐EPO cells, CD140b and CD73 should be used for sorting in further studies.

We proposed a modified differentiation protocol for hiPSC‐EPO cells. However, our study had some limitations. First, we did not investigate whether hiPSC‐EPO cells ameliorated renal anemia in CKD mice. Second, we did not measure the EPO concentration in low‐oxygen conditions. To show that the hiPSC‐EPO cells generated by our approach have the same function as EPO cells, further studies are needed. In conclusion, our results suggested that CD140b and CD73 are beneficial to cell surface markers for hiPSC‐EPO cells.

## Conflict of interest

KO is a founder and a member without salary of the scientific advisory boards of iPS Portal Japan. The other authors have no potential conflicts of interest.

## Author contributions

TM and HH conceived and supervised the study. SN, TM, and TN designed experiments. SN and FN performed experiments. AN, KT, YY, KO and TN provided new tools and reagents. TM and HH wrote the manuscript.
